# Toxicity Effect of Silver Nanoparticles on Mice Liver Primary Cell Culture and HepG_2_ Cell Line

**Published:** 2014

**Authors:** Firouz Faedmaleki, Farshad H Shirazi, Amir-Ahmad Salarian, Hamidreza Ahmadi Ashtiani, Hossein Rastegar

**Affiliations:** a Department of Basic Science, Science and Research Branch, Islamic Azad University, Tehran, Iran.; b Pharmaceutical Sciences Research Center, Shahid Beheshti University of Medical Sciences, Tehran, Iran.; c Deptartment of Toxicology , Aja University of Medical Sciense, Tehran, Iran.; d Islamic Azad University, Pharmaceutical Seines Branch, Tehran, Iran.

**Keywords:** Silver nanoparticle, HepG2 cell line, Primary liver cells of mice, MTT assay, Cell viability, Nanotechnology

## Abstract

Nano-silver (AgNP) has biological properties which are significant for consumer products, food technology, textiles and medical applications (e.g. wound care products, implantable medical devices, in diagnosis, drug delivery, and imaging).

For their antibacterial activity, silver nanoparticles are largely used in various commercially available products. Thus, the use of nano-silver is becoming more and more widespread in medicine.

In this study we investigated the cytotoxic effects of AgNPs on liver primary cells of mice, as well as the human liver HepG_2_ cell.

Cell viability was examined with MTT assay after HepG_2_ cells exposure to AgNPs at 1, 2, 3, 4, 5, 7.5, 10 ppm compared to mice primary liver cells at 1, 10, 50, 100, 150, 200, 400 ppm for 24h. AgNPs caused a concentration-dependent decrease of cell viability in both cells. IC_50_ value of 2.764 ppm (µg/mL) was calculated in HepG_2_ cell line and IC_50_ value of 121.7 ppm (µg/mL) was calculated in primary liver cells of mice. The results of this experiment indicated that silver nanoparticles had cytotoxic effects on HepG_2_ cell line and primary liver cells of mice. The results illustrated that nano-silver had 44 times stronger inhibitory effect on the growth of cancerous cells (HepG_2_ cell line) compared to the normal cells (primary liver cells of mice). which might further justify AgNPs as a cytotoxic agents and a potential anticancer candidate which needs further studies in this regard.

## Introduction

Nanotechnology and nanoparticles are increasingly recognized for their potential applications in aerospace engineering, nano-electronics, environmental remediation, medical healthcare and consumer products (1, 2, 3).

Nanoparticles, by definition, are structures that have dimensions in the range of 1–100 nm (4, 5).

With the rapid development of nanotechnology, nanoparticles applications have been extended further and now silver is the most commonly used engineered nano-material in consumer product (6, 10). In the field of medical applications, wound dressings, contraceptive devices, surgical instruments, bandages and bone prostheses are coated or embedded with nanosilver (20, 21, 24).Other uses of Silver nanoparticles are in respirators, household water filters, antibacterial sprays, cosmetics, detergent and textiles (3, 11, 12, 13, 14, 21, 22, 23).

Silver nanoparticle has antibacterial properties; action mechanism of silver nanoparticle on bacteria is demonstrated in different studies. Silver nanoparticles show efficient antimicrobial properties compare to other salts due to their extensive surface area which provide better contact with microorganisms. Silver nanoparticles attach to the cell membrane and penetrate into the bacteria. There are sulfur-containing proteins in bacterial membrane and silver nanoparticles interact with these proteins in the cell as well as with phosphorus containing compounds like DNA. When silver nanoparticles enter the bacterial cell forms a low molecular weight region in the center of bacteria where the bacteria conglomerates and protects the cellular DNA from the silver ions. Silver nanoparticles attack the respiratory chain and cell division that leads to the cell death. Silver nanoparticles release silver ions in the bacterial cells that enhance their bactericidal activity (36). Furthermore, their unique plasmon-resonance optical scattering properties allow AgNP use in bio-sensing and imaging applications (3).

More importantly, AgNPs showed potential in the treatment of diseases that require maintenance of circulating drug concentration or targeting of specific cells or organs (3). For example, AgNPs have been shown to interact with the HIV-1 virus and inhibit its ability to bind host cells *in-vitro* (3).Therefore, exposure to nano-silver in the body is becoming increasingly widespread and intimate. Consequently, silver in the form of nano-particles has gained an increasing access to tissues, cells and biological molecules within the human body.

Increasing evidence indicates adverse effects of NPs in human health as well as the environment. Their small size, high surface area per unit mass, chemical composition, and surface properties are important factors for their toxicities (7). Nonspecific oxidative damage is one of the greatest concerns for the use of nanoparticles (4, 8, 9 ). Despite their widespread application, comprehensive biologic and toxicologic information is insufficient. In addition, exposure and associated risk to human and environmental health have not been explored systematically, while there have been studies exploring the toxicity of metal NPs (15, 16), including Ag (17, 18). 

Cytotoxicity studies in human macrophages provided information on the toxicity of silver nanoparticles (14). Oral toxicity, genotoxicity, and gender-related tissue distribution of AgNPs in rats were also investigated (17). Subchronic inhalation toxicity of AgNPs was investigated which showed increases in lesions related to silver nanoparticle exposure (19, 25). The toxicity of AgNPs has been investigated in some cell types that illustrated toxicity of silver nanoparticles in those cells including BRL3A rat liver cells (4, 26), PC-12 neuroendocrine cells (4, 27), human alveolar epithelial cells (4, 28 ) and germline stem cells (4, ). However, direct evidence on toxic effects of unmodified AgNPs has not been fully documented at the cellular and molecular levels. Despite growing concerns, little is known about the potential impacts of AgNPs on human and environmental health.

In earlier studies Takenaka *et al*. (15, 20) reported that liver appeared to be a major accumulation site of circulatory silver nanoparticles. A recent clinical report also described absorption of nano-silver into the circulation following the use of nano-silver coated dressings for burns (30). In such cases, primary cells isolated from target tissues are desirable for cytotoxicity testing to simulate the in vivo situation more closely. Further, primary cultured liver cells (rodent or human origin) also represent a useful tool for studying toxicity, drug metabolism and enzyme induction (20, 32).

As a detoxifying organ, liver becomes particularly important when ingestion is the entrance route to the body. HepG2 (human hepatoblastoma) cell line may be used for xenobiotic metabolism studies as it maintains many specialized functions of normal liver parenchymal cells such as synthesis and secretion of plasma proteins and cell surface receptors. 

In this study, we investigated the toxic effects of AgNPs on human hepatoma derived cell line HepG2 as well as the primary hepatocyte of mice exposed to AgNPs at different doses. Toxicity evaluation and cell viability were assessed using MTT assay under exposed conditions and IC_50_ of silver nanoparticles was calculated on these cell cultures.

## Experimental

*Nanoparticle preparation*

AgNPs were provided by Nanonasb Pars Company. It was a clear colloidal aqueous suspension with a concentration of 4000 PPM (or mg/L) and particles size was 20-40 Nm. The particles could be stably diluted with distilled water (no color change and no precipitation), then this solution was diluted with deionized water and sterilized with microfilter (0.22 micron). 

*Cell culture*

The cell line HepG_2_ was obtained from National Center for Cell Sciences, Pasteur Institute of Iran (Tehran). The cells were cultured in RPMI-1640 medium supplemented with 1% (v/v) penicillin-streptomycin and 10% (v/v) heat inactivated FBS. Cells were maintained in 5% CO_2_ humidified incubator at 37ºC. During subculture, cells were detached by trypsinization when they reached 80% confluency. The well-grown cells were harvested and seeded into 96-well plates at a density of 15000 cells per well for experiments.

*Isolation and culture of primary mouse hepatocytes*

Swiss albino mice (7-10 day old) were euthanized with an intraperitoneal injection of ketamin and xylazine, and heparin was injected to them by intraperitoneal route; then mice were sterilized with 70% ethanol. For the isolation of liver cells, the excised liver was minced with a scalpel, and tissue fragments of liver were washed with Hanks buffer. Tissue particles were washed once with PBS (phosphate buffered saline) then tissue fragments of liver were transferred to solution of hanks with collagenase (17 mg collagenase in 25 mL hanks solution), incubated and agitated at 37 °C in shaker incubator for 2 hours. This solution of the cells were transferred to a 50 mL centrifuge tube and then were centrifuged for 10 minutes at 500 rpm. Supernatant was removed and the cells were re-suspended in complete medium (DMEM/F12) supplemented with 15% heat-inactivated fetal calf serum and 3% (v/v) penicillin-streptomycin. The viability of the hepatocytes was 95–100% as determined by Trypan Blue exclusion. Freshly isolated liver Cells of mice were seeded at a density of 15000 cells/well in 96-well plates. Wells were already coated with 15 microliter poly D-lysine. Cells were incubated in a humidified incubator at 37 ^◦^C containing 5% CO_2_ and 95% air for 24 hour.

*Cell count*

The human hepatocarcinoma HepG_2_ cell line and mice liver primary cell culture were harvested and seeded into 96-well plates at a density of 15000 cells per well for experiments. Cells were counted before and after being seed in the 96-well plates.

*MTT assay*

Cell viability was tested using MTT assay which was based on the cleavage of the tetrazolium salt (MTT) by metabolically active cells to form a formazan dye that was water-insoluble. The insoluble dye formed in MTT assay was solubilized using DMSO. The MTT assay was performed according to a modification of the method described by Mosmann (1983). Cells were seeded in 96-well tissue culture plates (15000 cell/well in 100 µL culture medium, RPMI1640 for HepG_2_ cell line ,DMEM/F12 for primary liver cells of mice) and incubated overnight at 37 ^◦^C and 5% CO_2_, then morphology of cells were observed in invert microscope before exposure to silver nano-particles. After overnight growth, supernatants in the culture plates were aspirated out and then 10µL silver nanoparticles solutions that diluted with deionized water were added in concentrations 1, 2, 3, 4, 5, 7.5, 10 ppm to HepG_2_ cell line and 1, 10, 50, 100, 150, 200, 400 ppm to primary liver cells of mice in each well of 96-well tissue culture plates. To grow cells 90 µL of culture medium (RPMI1640 for HepG_2_ cell line and DMEM/F12 for primary liver cells of mice) was added to each well. Treated cells were incubated for 24 hour at 37 ^◦^C and 5% CO_2_. Morphology of cells was observed in invert microscope after exposure to different concentrations of silver nanoparticles. After overnight, supernatants were aspirated out and 10 µL MTT solution (50 mg/10 mL PBS) was added to each well then 90 µL of culture medium (RPMI1640 for HepG_2_ cell line and DMEM/F12 for primary liver cells of mice) was added to each well and the plates were incubated for 4 h in the case of HepG_2_ cell line and 6 h for the primary liver cells of mice. Supernatants were replaced by100 µL DMSO and plates were shaken at 37 ^◦^C in shakerincubator for 15 minutes then absorbance at two wavelengths (570 nm and 650 nm) was recorded using ELISA reader. All absorbance values were corrected against blank wells which contained growth media alone. Each assay involved six wells per condition.

*Statistical analysis*

All experiments were carried out in triplicates, independently. The data obtained were expressed in terms of ‘mean ± standard deviation’ values. Wherever appropriate, the data were also subjected to one-way ANOVA using with software GraphPad Prism 5. The value of p < 0.0001was considered as significant.

## Results

**Figure 1:**
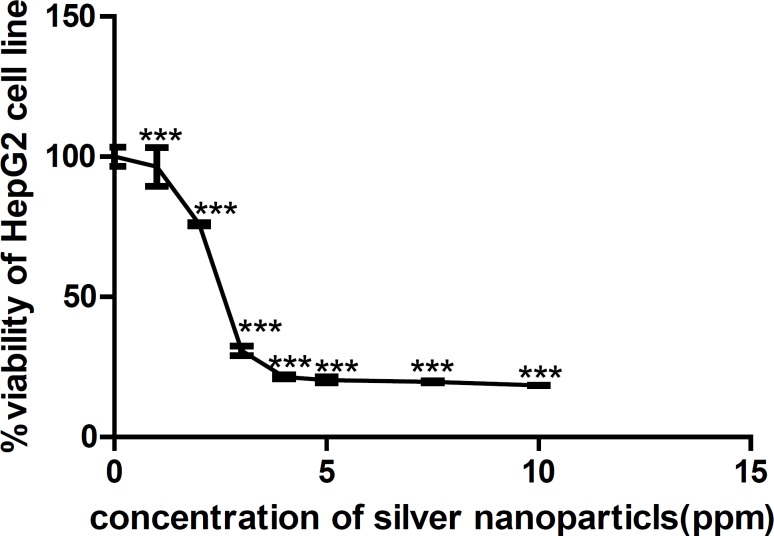
Viability percentage measured by MTT assay on HepG2 cell line exposed to 0, 1, 2, 3, 4, 5, 7.5, 10ppm of AgNPs for 24 h. An OD value of control cells (unexposed cells) was taken as 100% viability (0% cytotoxicity). Data were reported as mean ± SD of three independent experiments performed in quadruplicate. The relative cell viability related to control was calculated by [OD] test/[OD] control **×**100. Using the dose-response curves, IC_50_ was calculated to be 2.764 µg/mL (ppm). (***p < 0.0001).

**Figure 2:**
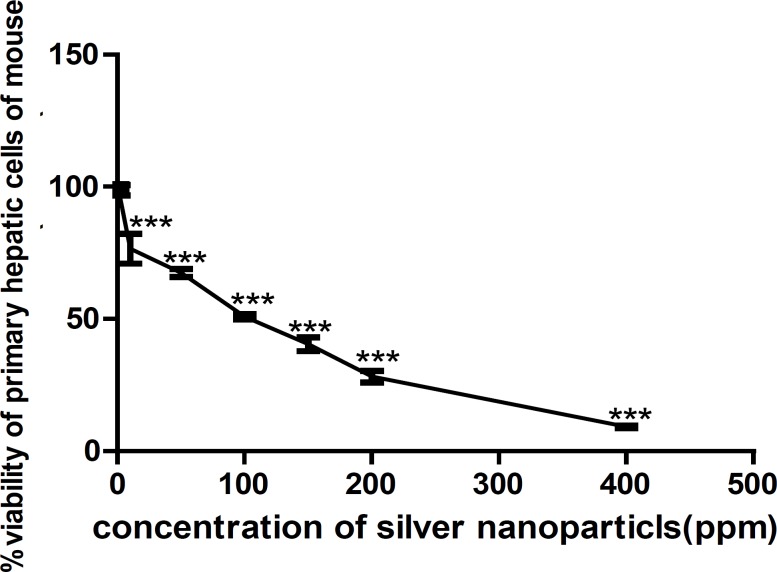
Viability percentage measured by MTT assay on primary liver cell of mice exposed to 0, 1, 10, 50, 100, 150, 200, 400 ppm of AgNPs for 24 h. An OD value of control cells (unexposed cells) was taken as 100% viability (0% cytotoxicity). Data were reported as mean ± SD of three independent experiments performed in quadruplicate. The relative cell viability related to control was calculated by [OD] test/ [OD] control **×**100. Using the dose-response curves, IC_50_ was calculated to be 121.7 µg/mL (ppm).(*** p < 0.0001).

The results of MTT assays showed a dose-dependent decrease in viability percentage of both HepG_2_ cell line and the primary liver cells of mice after 24 h exposure to AgNPs (Figure 1, 2). 

Viability percentage measured by MTT assay on HepG_2_ cell line exposed to 0, 1, 2, 3, 4, 5, 7.5, 10 ppm of AgNPs for 24 h represented a dose-response pattern as shown in Figure 1. Data were reported as mean ± SD of three independent experiments performed in quadruplicate. Using the dose-response curves, IC_50_ was calculated to be 2.764 µg/mL (ppm). (p < 0.0001).

Viability percentage measured by MTT assay on primary liver cell of mice exposed to 0, 1, 10, 50, 100, 150, 200, 400 ppm of AgNPs for 24 h represents a dose-response pattern as is shown in Figure 2. Data were reported as mean ± SD of three independent experiments performed in quadruplicate. Using the dose-response curves, IC_50_ was calculated to be 121.7 µg/mL (ppm) for AgNP on HepG_2_ cell line. (p < 0.0001).

The relative cell viability related to control was calculated by [OD]test/[OD]control **×**100. Using the dose-response curves, IC_50_ was calculated to be 121.7 µg/mL (ppm). (*** p < 0.0001).

**Figure 3:**
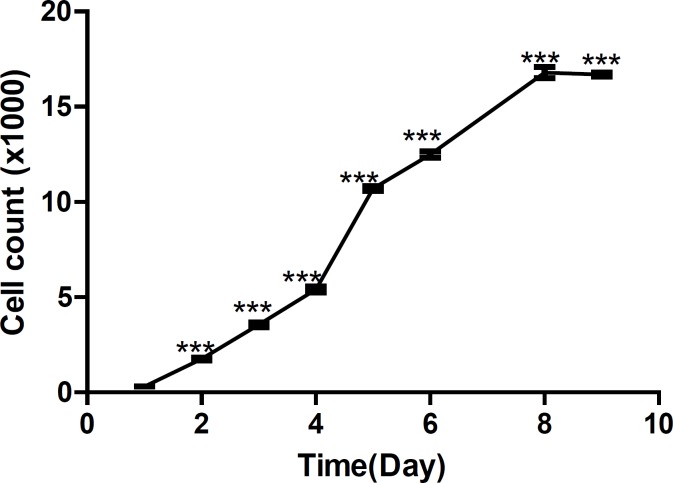
Growth curve of HepG_2_ cells. (***p < 0.0001)

**Figure 4:**
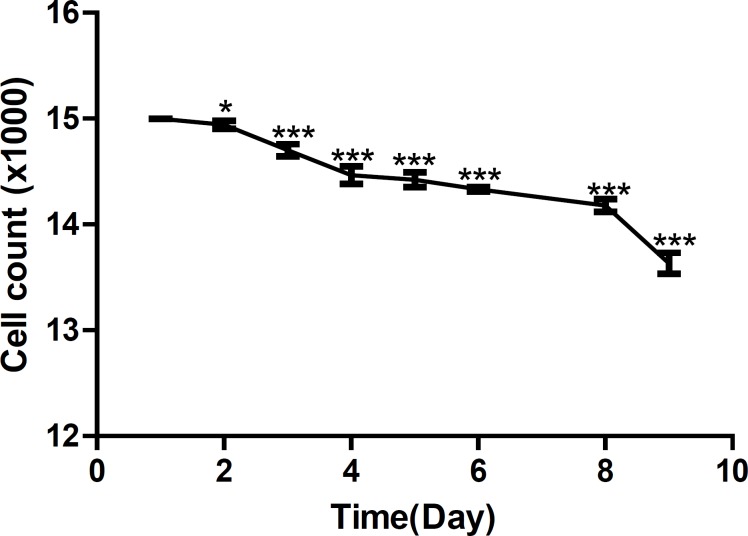
Growth curve of mice liver primary cell culture.

Figures 3 shows growth curve of HepG_2_ cells and Figure 4 shows growth curve of mice liver primary cell culture. Figure 3 also shows an increase of growth in cell line compare to the primary cells in Figure 4. However, as is shown in these Figures, the first three days of growth follows the same pattern.

(*p 0.01 to 0.05,***p < 0.0001)

*Effects of Ag NPs on cellular morphology*

Figure 5 shows morphological changes of HepG_2_ cells exposed to Ag NPs at 1 ppm and 10 ppm and primary liver cells of mice exposed to AgNPs at 120 ppm for 24 h using inverted microscope. Significant morphological changes of cell death, including restricted spreading patterns and increased floating cells were observed in HepG_2_ cells exposed to different concentrations of Ag NP and primary liver cells of mice exposed to AgNPs at 120 ppm.

**Figure 5a:**
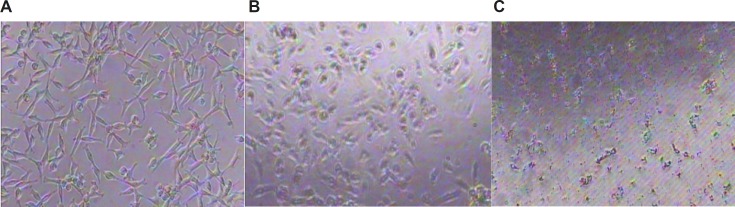
Morphological characterization of HepG_2_: A: untreated HepG_2_ cell line, B: treated HepG_2_ cell line at 1 ppm of AgNP for 24 h, C: treated HepG_2_ cell line at10 ppm of AgNP for 24 h

**Figure 5b:**
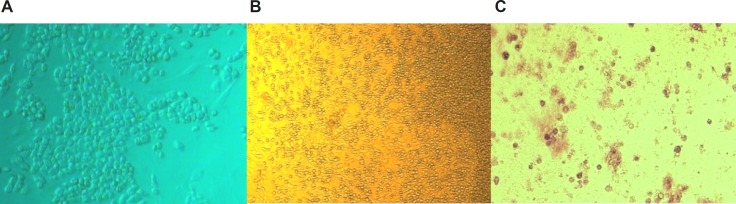
Morphological characterization of primary liver cells of mice: A: untreated primary liver cells of mice, B: untreated primary liver cells of mice with high confluency, C: treated primary liver cells of mice at 120 ppm of AgNPs for 24 h

## Discussion

Surface reactivity, chemical composition, and large specific surface area have been deemed important properties in NP-mediated toxicity (7). Due to their physicochemical properties, it is likely that AgNPs have unique mechanisms of toxic actions.

Cytotoxicity induced by silver nanoparticles and the role that oxidative stress plays in this process were demonstrated in human hepatoma cells AgNPs agglomerated in the cytoplasm and nuclei of treated cells, and they induced intracellular oxidative stress. Previous findings have been suggested that AgNP cytotoxicity was primarily as the result of oxidative stress and independent of the toxicity of Ag^+ ^ions (4).

It has also been shown that AgNP reduced ATP content of the cell and caused damage to mitochondria and increased production of reactive oxygen species (ROS) in a dose-dependent manner. The nanoparticle treatment caused cell cycle arrest in G_2_/M phase possibly due to repair of damaged DNA (3, 13, 33 ).

Once nanoparticles are absorbed by the gastrointestinal tract, they will be transported directly to the liver via the portal vein. In general, the liver is able to actively remove compounds from the blood and transform them to chemical forms that can easily be excreted.

Uptake of nano-silver by the liver and subsequent excretion in the bile represents another possible excretion route. After inhalation of nano-silver particles of 12.6-15.3 nm (15).

*In-vitro* exposure of human peripheral blood mononuclear cells (PBMCs) to silver nanoparticles (1-2.5 nm, 72 h) resulted in the inhibition of phytohemagglutinin (PHA) induced proliferation (at a concentration 15 ppm) (34). 

Hussain *et al.* (26) evaluated the in vitro toxicity of several nanoparticles, including nano-silver (15 and 100 nm) on a rat liver derived cell line (BRL 3A). Following 24 h after exposure the mitochondrial function and membrane integrity (measured as LDH leakage) were significantly decreased (at 5 mg/mL and 10 mg/mL, respectively). LDH leakage was dose dependent and more severe for 100 nm than for 15 nm silver nanoparticles. Visual microscopic evaluation indicated that not all nanoparticles accumulated in the cell, but some remained associated with membranes. All other tested nanoparticles (Fe_3_O_4_, Al, MoO_3_, MnO_2_) appeared to be less toxic than nano-silver. The observed cytotoxicity was attributed to be mediated by oxidative stress, as indicated by the detection of GSH depletion, reduced mitochondrial potential, and increased reactive oxygen species (ROS) levels. A similar concentration-dependent cytotoxicity was observed when the effects of the same nano-silver particles on a mouse cell line with spermatogonial stem cell characteristics was studied (29). 

In contrast, adding of 1.0% silver nanoparticles (5-50 nm) to bone cement, a dose at which antibactericidal activity was seen, did not result in (additional) cytotoxicy towards mouse fibroblasts (L929), or on-growth of human osteoblast cell line (35).

In HaCaT cells 24 h exposure to AgNPs caused reduction of mitochondrial function, as measured by the MTT assay, at the highest concentrations tested (11 and 36 µg/mL). Similar results have been reported investigating the effect of AgNPs on different cell lines, such as human (0.5–3 µg/mL) and rat (10–50 µg/mL) liver cells, alveolar macrophages (10–75 µg/mL), mice dermal fibroblasts and liver cells (30 µg/mL) or mouse germline stem cells (10µg/mL) (4, 26, 31, 20, 29).

With regard to the mechanisms by which AgNPs can affect cell viability, in contrast to the relatively abundant studies showing evidence about the induction of oxidative stress and apoptosis, only few studies have investigated the intracellular pathways involved in these processes. 

## Conclusion

The results of this study demonstrated that silver nanoparticles was a toxic agent at high doses on HepG_2_ cell line and primary cell culture of mice. The results illustrated that nano-silver had 44 times more inhibition effect on the growth of cancerous cells (HepG_2_ cell line ) as compared to the normal cells (primary liver cells of mice). Further cellular and molecular investigations for a better understanding of involved mechanisms might potentiate AgNPs application as anticancer drugs.
